# Design of an intelligent controller for a grain dryer: A support vector machines for regression inverse model proportional–integral–derivative controller

**DOI:** 10.1002/fsn3.1340

**Published:** 2020-01-20

**Authors:** Aini Dai, Xiaoguang Zhou, Zidan Wu

**Affiliations:** ^1^ Science and Information College Qingdao Agricultural University Qingdao China; ^2^ School of Economics and Management Minjiang University Fuzhou China; ^3^ School of Automation Beijing University of Posts and Telecommunications Beijing China

**Keywords:** genetic algorithm, grain drying, indirect inverse model controller, support vector regression

## Abstract

Grain drying control is a challenging task owing to the complex heat and mass exchange process. To precisely control the outlet grain moisture content (MC) of a continuous mixed‐flow grain dryer, in this paper, we proposed a genetically optimized inverse model proportional–integral–derivative (PID) controller based on support vector machines for regression algorithm which is named the GO‐SVR‐IMCPID controller. The structure of the GO‐SVR‐IMCPID controller consists of a genetic optimization algorithm, an indirect inverse model predictive controller, and a PID controller. In addition, to verify the control performances of the proposed controller in the simulation study, we have established a nonlinear mathematical model for the mixed‐flow grain dryer to represent the nonlinear grain drying process. Finally, the control performance and the robustness of the GO‐SVR‐IMCPID controller were simulated and compared with the other controllers. By the simulation results, it is shown that this proposed algorithm can track the target value precisely and has fewer steady errors and strong ability of anti‐interference. Furthermore, it has further confirmed the superiority of the proposed grain drying controller by comparing it with the other controllers.

## Graphical abstract

An intelligent controller (GO‐SVR‐IMCPID) based on support vector machines for regression modeling method for a grain dryer is proposed. The GO‐SVR‐IMCPID has good tracking control performance, strong ability of anti‐interference, and good robustness performance by control simulation result comparison with other controllers.

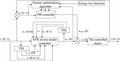

## 1 INTRODUCTION

Studying the grain drying control is significant (Dai, Zhou, & Zhou, 2017; Dai, Zhou, Liu, Liu, & Zhang, 2017, 2018; Liu & Arkema, 2001; Liu & Bakker‐Arkema, 2001; Mujumdar, 1995).Grain drying control is a challenging task owing to the complex heat and mass exchange process. It is such a long‐delay nonlinear process subjected to various affection factors that an accurate mathematical model is difficult to make. Hence, the classical traditional controllers have some limitations in the control of grain drying and effective control strategies need to be further researched on.

Traditional control methods mainly include feedback control, forward control, forward feedback control, and other classical traditional control methods. These methods are usually designed based on linear transfer function and cannot deal with the changes of complex nonlinear system. Some scholars have studied the limitations of traditional control algorithms in the grain drying control (Liu & Arkema, 2001; Liu & Bakker‐Arkema, 2001). Proportional–integral–derivative (PID) controller is successfully applied in the classic automatic control, but the control effect of which in the grain drying is not satisfactory because the controlled drying object and the environment are uncertain, and it cannot satisfy the control requirement of grain drying with stricter restriction on drying control performance (Lutfy, Selamat, & Noor, 2015). Model predictive control (MPC) is effective for the long‐delay nonlinear control system. Liu et al. have presented a MPC which was especially designed to control a cross‐flow dryer. Field testing and simulation both showed the MPC performed excellence in accuracy, stability, and robustness (Liu & Arkema, 2001; Liu & Bakker‐Arkema, 2001). However, the traditional MPC still depends on the system model, which is usually simplified on the basis of some assumptions. When using these models for control purposes, these simplifications will affect the control accuracy to a certain extent. The partial differential equations (PDE) of grain drying have good universality and wide application, but they are too complex to be used to carry out the real control because it will take more computing time to solve the complex equations and boundary conditions (Lutfy et al., 2015). Aiming at the difficulty of modeling the complex grain drying process, intelligent identification methods such as Fuzzy logic (FL) algorithms methods or artificial neural network (ANN) are best adopted to approximate the nonlinear relationship, which can be used to construct controllers by combining MPC control, adaptive control, sliding mode control, backstepping control, and evolutionary algorithms (EA) or their combinations (Wang, Sun, & Liu, 2017).

Artificial neural network has a wide range of applications in drying control, which is an excellent tool to model complex, dynamic, highly nonlinear, unclear scientific and engineering problems (Aghbashlo, Hosseinpour, & Mujumdar, 2015; Çakmak & Yıldız, 2011; Dai, Zhou, & Zhou, 2017; Dai et al., 2017, 2018; Farkas, Remenyi, & Biro, 2000; Li & Chen, 2019; Li, Xiong, Wang, & Shi, 2016; Movagharnejad & Nikzad, 2007; Tsai & Luo, 2017). Besides ANN, support vector machines (SVM) for regression is also a promising approach to model the grain drying, which is superior to ANN, including generalization ability, ease of training, a mechanism to model structured data, and, most importantly, the generation of a unique solution (Hou & Zou, 2016), However, ANNs are confronted with the problems of trapping local minima, slow learning, and metaparameter adjustment (Patil & Deka, 2016). Furthermore, compared with the traditional learning method based on large samples, SVM is more suitable for learning based on small samples (Colman, Waegeman, & Baets, 2015), so modeling the complex system with its excellent approximation ability would be a better way. Now, there are some relevant control literatures that use support vectors in control approximation (Chakrabarty, Buzzard, & Zak, 2017; Chakrabarty et al., 2017; Wei et al., 2017). However, there is not much research on SVM control of grain dryer in the literature.

The support vector machines for regression (SVR) control methods mainly include the inverse model control of SVR, the internal model control of SVR, and the SVR optimal control. In another study (Dai, Zhou, & Zhou, 2017; Dai et al., 2017, 2018), we have designed a genetically optimized internal model PID predictive controller (GO‐SVR‐IMPC) which has proved to be effective in controlling the grain drying process. To further study the SVR controller for the grain dryer, in this paper, we proposed a SVR indirect inverse model controller and compared it with different controllers including the previously designed GO‐SVR‐IMPC controller.

The inverse model controller of SVR (SVR‐IMC) is an effective open‐loop control method, which is based on the inverse dynamic theory. A pseudolinear system is formed by connecting the indirect inverse model and the controlled object in series, and thus, a nonlinear controller can be further designed using the linear control theory. Because the open‐loop control structure of SVR‐IMC cannot handle the effect of the external disturbances, it is often improved by combining the other methods to form a closed‐loop control structure in actual control, of which the most effective improvement method is to introduce feedback control to form a closed‐loop system. In this paper, a genetically optimized PID feedback controller based on a support vector inverse model algorithm (GO‐SVR‐IMCPID) for a field mixed‐flow grain dryer is investigated to improve the control deficiency of direct inverse model controller.

This paper's contributions are as follows: In view of the modeling advantage of SVR that would be better on dealing with the highly nonlinear process of engineering based on small samples, a SVR‐IMC is designed, which is a simple and an effective open‐loop control method; and then, a genetically optimized PID feedback control algorithm is introduced to the SVR‐IMC to form a closed‐loop controller, and the controller performance is optimized by establishing a performance objective function of the grain drying control from decreasing the energy consumption and improving the dried grain quality; finally, the effectiveness of the GO‐SVR‐IMCPID has been verified in the control simulations by using a nonlinear mathematical drying model to represent the practical mixed‐flow drying process. The simulations and comparisons of the control performance have proved the superiority of the controller designed in this paper. It is a better choice to control the complex grain drying process.

The rest of this article is arranged as follows: A novel grain drying system and the proposed mathematical model for the mixed‐flow grain drying process are given in Section 2. In the third section, a genetic optimization inverse model PID controller based on SVR is proposed to control our designed grain drying system. Section 4 introduces and analyses the results of the control simulations and control performance comparison. Finally, the work has been concluded in Section 5.

## 2 A NOVEL GRAIN DRYING SYSTEM

### 2.1 Experimental system

**Figure 1:**
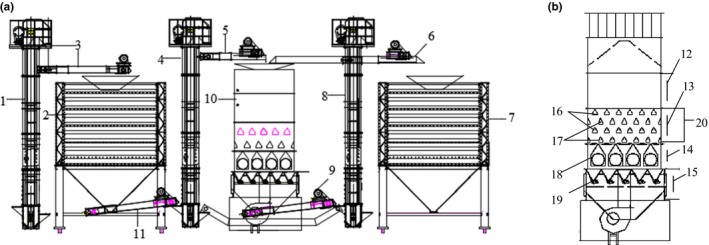
(a) The structure diagram: the mechanical system of the grain drying system. (1, 4, and 8: bucket elevators; 2: wet grain bin; 3, 5, 6, 9, and 11: belt conveyors; 7: dried grain bin; 10: dryer; 12: storage section; 13: convection section; 14: radiation section; 16: the inlet wind angle box; 17: the outlet wind angle box; 18: combustion chamber; 19: grain discharging wheel; 20: the waste gas room.) (b) The structure diagram: the 5HSHF10‐type grain dryer

**Figure 2:**
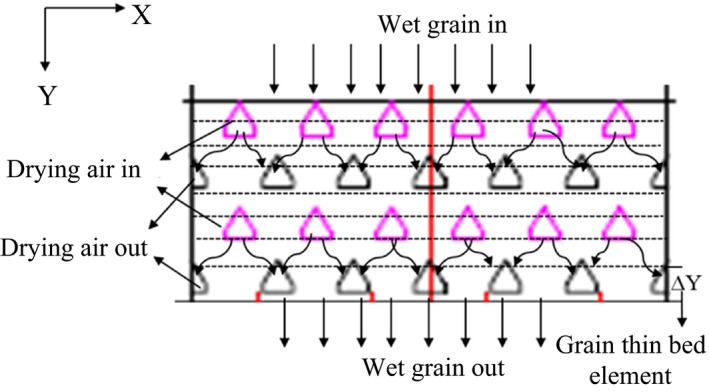
A simplified scheme of the mixed‐flow drying

Figure 1a shows the designed combined multifunctional grain drying system. It mainly consists of a wet grain bin for storing wet grains, a 5HSHF10 dryer for heating and drying grains, and a dried bin for storing dried grains. The other part is composed of five belt conveyors, three bucket elevators, etc. The 5HSHF10‐type grain dryer is shown in Figure 2, and its specific parameters can refer to Table 1. Figure 1b shows the 5HSHF10‐type dryer whose shape is rectangular and shape size is 4.75 m high, 2.06 m long (thick), and 1.3 m deep (Dai, Zhou, & Zhou, 2017; Dai et al., 2017, 2018). The convection section of the grain dryer is a combination design, and there are three kinds of drying technique to choose by replacing the drying section. In this paper, we mainly discuss the control of the continuous mixed‐flow grain drying process. The process of the continuous mixed‐flow drying technology is as follows: the wet grains flow into the dryer from the top of the dryer and then pass through the mixed‐flow drying section with hot air angle box, the radiation section, and the discharging grain section in sequence. Finally, according to the set grain flow rate, the final grain moisture content (MC) value is determined by the outlet grain MC of the dryer, which is evacuated from the bottom of the mixed‐flow drying section at discrete variable time intervals. As shown in Figure 2, hot air angle box can be input and output flow arrangement alternately and the drying hot air is blown into the dryer from the inlet angle box and blown out from the outlet angle box. Because the wind in the dryer has the direction of concurrent‐flow, counter‐current, and cross‐flow, it is named mixed‐flow drying technology. Because of its continuous mixed‐flow effect, it will produce a good dried grain quality.

**Table 1:** Parameters and expression of grain in the simulation experiment

Parameters	Symbol	Value or expression	Unit
Initial hot air temperature	*T*_1_	100	°C
Hot air temperature	*T*_a_	/	°C
Initial MC of wheat	*M*_1_	0.23	Decimal, wet basis (wb).
The height of a thin element	∆*Y*	0.05	m
Drying coefficients of wheat	*k*_1_	$\mu *e^{-r/(1.8*\left(T_{1}+273\right))}$	/
Drying coefficients of wheat	*μ*	1,941	/
Drying coefficients of wheat	*r*	5,032	/
Exponent *α* for wheat	*α*	−20.4 + 0.075*T*_1_	/
Exponent *β* for wheat	*β*	1,2522–37.3*T_1_	/
The grain density	*ρ*_g_	660	kg (wet matter)/m^−3^
The specific heat of dry air	*C*_a_	1.007	kJ/kg^−1^
The heat of vaporization	*h*_g_	2,427 + 2.17*35*(*M_i_*−0.183)	kJ/kg^−1^
The airflow rate	*V*_a_	43,200	kg (wet matter) hr^−1 ^m^−2^
The grain flow rate	*V*_g_	4,300	kg (wet matter) hr^−1 ^m^−2^

In the grain drying engineering, the discharging grain speed is usually manually operated by a technical worker with a long‐term drying experience, which is a time‐consuming and energy‐consuming job. To effectively control the dryer, an intelligent control algorithm is designed and simulated to automatically control the discharging grain speed in this paper. The controlled variable is the average outlet grain MC which is on‐line measured by a capacitive sensor, the control variable is the grain flow rate between two sampling intervals, and other affection factors are assumed as the disturbance signals. In addition, energy consumptions and dried grain quality have been also considered in this controller design.

### 2.2 The mathematical model of the dryer

**Figure 3:**
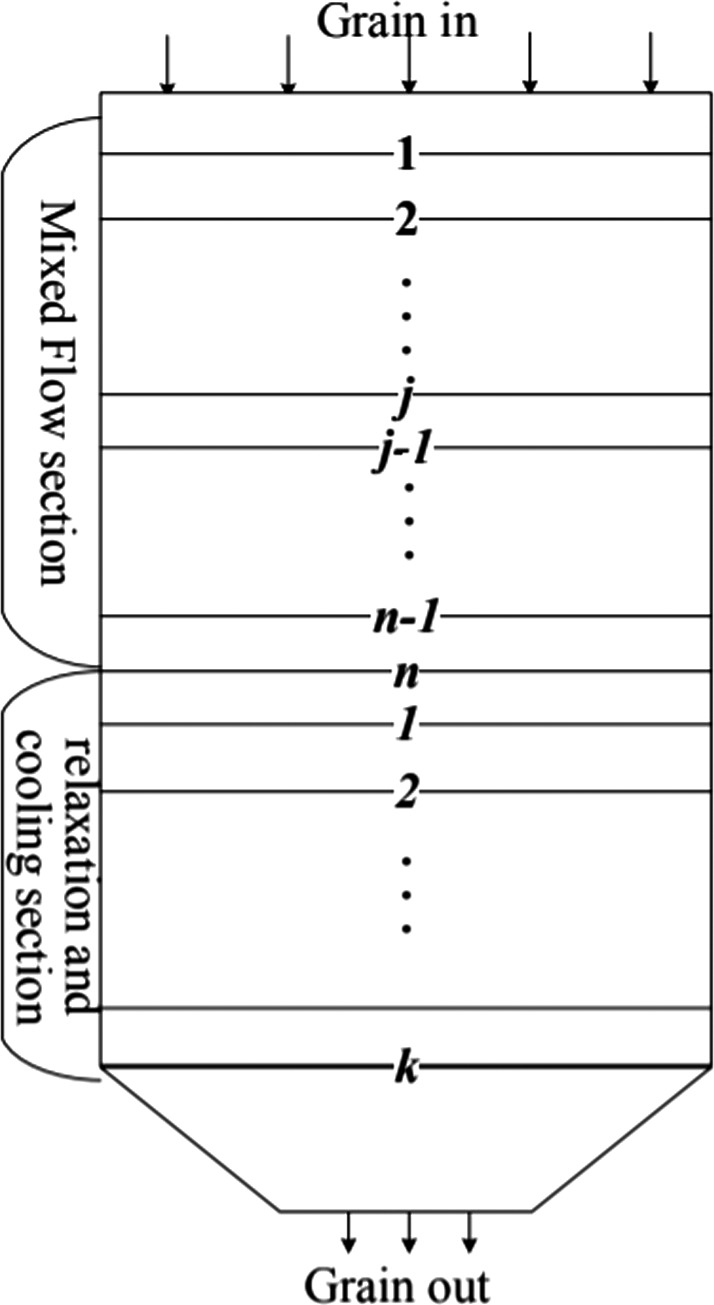
The continuous mixed‐flow grain dryer model

A mathematical model for the mixed‐flow drying process has been designed and introduced in detail in our previously published research paper (Dai, Zhou, & Zhou, 2017; Dai et al., 2017, 2018). As shown in Figure 3, in the continuous mixed‐flow grain dryer model, the grain column is divided into a series of thin layers and the drying time is divided into a series of intervals, wherein the drying section is divided into *n* beds, the cooling section is divided into *k* beds, and the height ∆*Y* shown in Figure 2 is very small, so the variation of air temperature and humidity in the *Y* direction of the element can be neglected. The ∆*Y* is equal to 0.1 m; according to the size of the novel dryer, *n* and *k* are equal to 20 and 30, respectively. The mixed‐flow dryer model is the collection of *n*‐column thin bed element models of the drying section in the grain dryer.

**Figure 4:**
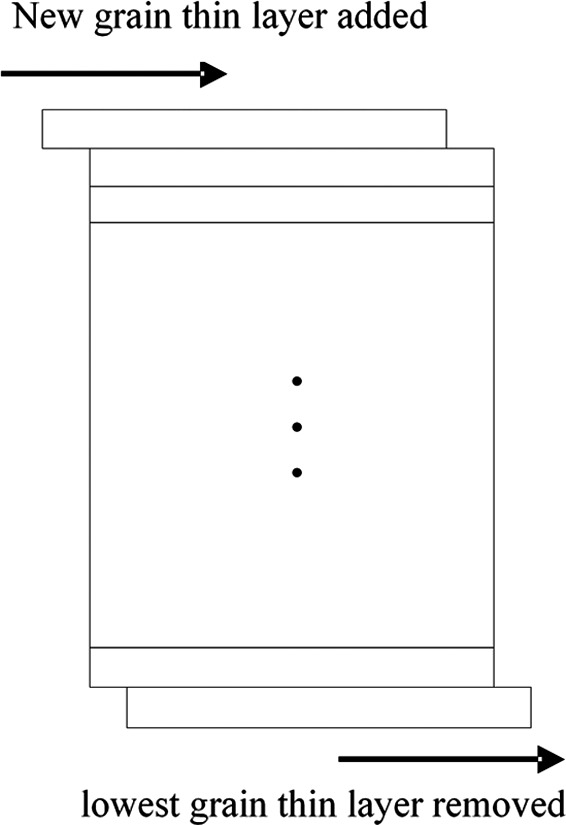
The calculation model at the next ∆*t* time interval

The calculation steps of the dryer model are as follows: step 1: In the drying section with *n* thin layers, the drying airflows through each layer in turn, we can use the existing thin bed element equation to calculate the next thin bed element equation; thus, at the ∆*t* time interval, the MC distribution in the grain dryer can be calculated in turn according to the Equation (3); step 2: As shown in Figure 4, at the next ∆*t* time interval, the lowest grain thin layer was removed, and a new thin layer was added to the original location of the first grain thin layer, and then, the MC distribution in the drying section is calculated again in light of step 1. Thus, step 1 and step 2 are repeated continuously, and the MC distribution of the grain dryer at each drying time can be worked out.

In (1), assuming that the heating of grain can be neglected, the drying process is regarded as an iso‐enthalpy process, and the initial drying parameters are shown in Table 1. ${\bar{M}}_{j-1}$: MC of the (*j *− 1)th thin bed element, ${\bar{M}}_{j}$: the MC of the *j*th thin bed element.

(1) $${\bar{M}}_{j}={\bar{M}}_{j-1}-\frac{k_{1}\left({\bar{M}}_{j-1}-M_{e1}\right)\times \Delta Y}{V_{g}\left(\frac{1}{\rho_{g}}+k_{1}{\bar{M}}_{j-1}c_{1}\gamma -k_{1}M_{e1}c_{2}\gamma \right)}$$

where

$$k_{1}=\mu *e^{-r/(1.8*\left(T_{1}+273\right))};\;$$

$$M_{e1}=0.01e^{\alpha +\beta /(1.8*\left(T_{1}+273\right))};$$

$$\alpha =-20.4+0.075T_{1};$$

$$\beta =12522-37.3T_{1};$$

$$\gamma =\frac{h_{g}\left(0.9\Delta Y\right)}{c_{a}V_{a}};\;$$

$$c_{1}=\frac{1-\exp\left(r/1.8(T_{1}+273)\right)-r/618}{1.8(T_{1}+273)-618};\;$$

$$c_{2}=\frac{1-\exp\left(\left(r-\beta \right)/1.8\left(T_{1}+273\right)\right)-\left(r-\beta \right)/618}{1.8(T_{1}+273)-618}$$

By taking the outlet MC of the (*j *− 1)th thin bed element ${\bar{M}}_{j-1}$ as the initial MC of the jth thin bed element, the MC change of the j^th^ element can be calculated according to the above Equation (1). By using the Equation (1) *n* times, the whole grain moisture distribution of the drying section can be obtained.

**Figure 5:**
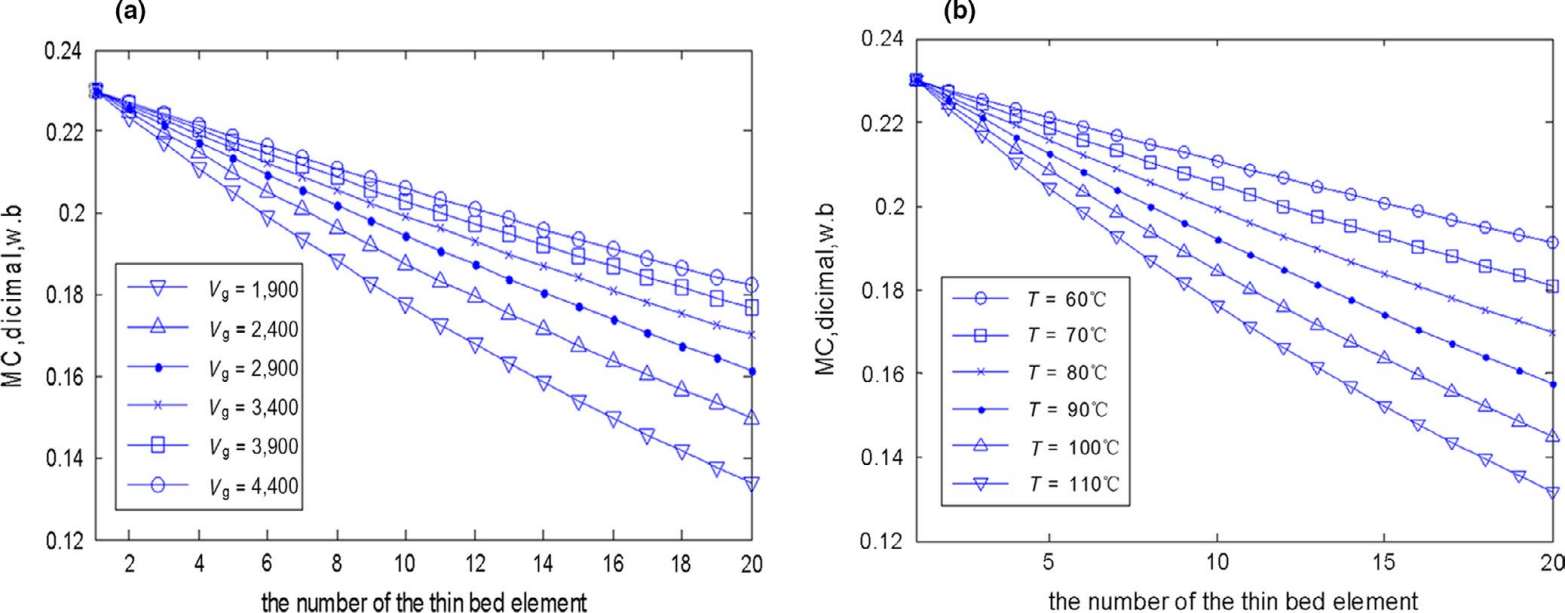
(a) The grain moisture content (MC) distribution of the continuous drying at the drying section: under different grain flow rate (unit: kg hr^−1^ m^2^) at *T*_1_ = 100°C. (b) The grain MC distribution of the continuous drying at the drying section: under different hot air temperature (unit: °C) when *V*_g_ is equal to 2,400 kg hr^−1^ m^−2^

**Figure 6:**
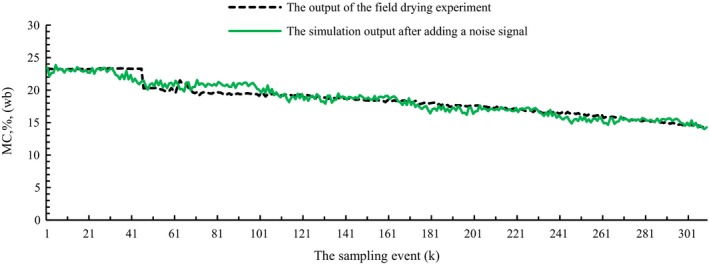
The outlet grain moisture content comparison

The above continuous drying model of the grain dryer was simulated in MATLAB. Figure 5a shows that different grain flow rates affect the MC distribution of grain, and the faster the grain flow rate is, the higher the grain MC of the same bed element at the drying section is. Figure 5b shows that the lower the hot air temperature is, the higher the grain MC of the same bed element at the drying section is. In real grain drying control, the hot air temperature is usually set to a constant, the target value of the outlet grain MC will be obtained by controlling the discharging grain flow rate of the dryer. As shown in Figure 6, the comparison with the field batch circulating drying experiment results depicts that the model has achieved a better modeling accuracy and more information about this model can refer to the literature (Dai, Zhou, & Zhou, 2017; Dai et al., 2017, 2018). Hence, the designed dryer model can be used to verify the feasibility of the designed controller of this paper.

## 3 DESIGN OF THE GO‐SVR‐IMCPID CONTROLLER FOR THE GRAIN DRYING SYSTEM

This part mainly introduces the inverse model control theory based on SVR. The basic idea of IMC‐SVR is to design an inverse model of the controlled drying process, and the feasibility of this method depends on the accuracy of the inverse model to a great extent. Compared with the ANN methods, SVR shows obvious superiority in this respect. So the SVR modeling method is introduced to design an inverse model for the controlled system to implement the identity map between the desired output of the system and the actual output.

### 3.1 Principle of SVR

Support vector machines for regression is a new machine learning method proposed by Vapnik et al., which is based on statistical learning theory and structural risk minimization (SRM) principle. It was firstly developed to solve classification tasks and then was used to solve regression tasks which is called support vector machines for regression (SVR). SVM can solve nonlinear, small‐sample, and high‐dimensional problems and has been widely used in the last decades (Alonso & Bahamonde, 2013; Rajaee & Boroumand, 2015; Smola & Schölkopf, 2004). It can map the input space to a high‐dimensional feature space by using a kernel function and performs well for regression problems. The complexity of computation depends on the number of support vectors rather than the dimension of the sample space, which avoids the curse of dimensionality in some sense.

Suppose a set of training data is given as $\left\{\left(x_{i},y_{i}\right)|x_{i}\in R^{n},y_{i}\in R^{l}\right\},i=1\ldots \ldots m,$ where $x_{i}$ is the input and $y_{i}$ is the target output, the purpose of regression with SVR is to find a function *f*(*x*) to fit all the sampling data, as shown in (2), which is close to the target value $y_{i}$. The optimal hyperplane that SVR seeks is not to divide the two classes to the most extent, but to minimize the total variance of the sample point from the hyperplane.

(2) $$f\left(x\right)=w^{T}\phi \left(x\right)+b\text{.}$$

Among them, *ϕ*(*x*) is the mapping form of vector *x*, which maps vector *x* to the high‐dimensional Euclidean space of *X* = *ϕ*(*x*). $w\in R^{n}$ and b∈R are the model parameters to be determined.

The ε‐SVR method uses an insensitive loss function which has at most ε deviation from the actually obtained targets *y_i_* for all training data. This is equivalent to build a width zone of 2ε of the interval by taking *f*(*x*) as the center. It is considered that the prediction values that fall into the interval band are correct. Thus, the SVR primal problem can be formalized as (3):

(3) $$\min\frac{1}{2}\left|\right|w^{2}\left|\right|+\delta \sum_{i=1}^{m}l_{\varepsilon }\left(f\left(x_{i}\right)-y_{i}\right)$$

where δ is the penalty factor on samples out of error‐*ε*, and the function of ε‐insensitive loss is shown in (4):

(4) $$l_{\varepsilon }\left(f\left(x_{i}\right)-y_{i}\right)=\left\{\begin{array}{cc} 0, & if|f\left(x_{i}\right)-y_{i}|\leq \varepsilon \\ \begin{array}{c} |f\left(x_{i}\right)-y_{i}|-\varepsilon , \end{array} & \text{otherwise} \end{array}\right.$$

The kernel function technology is adopted in the SVR model shown in (5).

(5) $$f\left(x\right)=\sum_{j=1}^{n}\left(\alpha_{j}^{*}-\alpha_{j}\right)K\left(x,x_{j}\right)+b=\sum_{j=1}^{n}\lambda_{j}K\left(x,x_{j}\right)+b$$

where $\lambda_{j}=\alpha_{j}^{*}-\alpha_{j}\left(0\leq \alpha_{j},\alpha_{j}^{*}\leq \delta \right)$, $\lambda_{j}$ is the weight coefficient of the support vector, $x_{j}$ is the support vector, and *n* is the number of support vectors.

The result is equivalent that the kernel function *K*(*x*, *x_j_*) enable operations to be performed in the input space rather than in the high‐dimensional feature space.

In this paper, the radial basis function (RBF) is adopted as the kernel function which has fewer required parameters and can handle the nonlinear relationship well shown in (6).

(6) $$K\left(x,x_{j}\right)=\exp(-\left|\right|x-x_{j}{\left|\right|}^{2}/\left(2*\gamma^{2}\right).$$

From the above analysis, it can be seen that three parameters:ε, δ, and γ need to be first determined in the SVR model.

### 3.2 Design of the inverse model controller based on SVR (IMC‐SVR)

**Figure 7:**
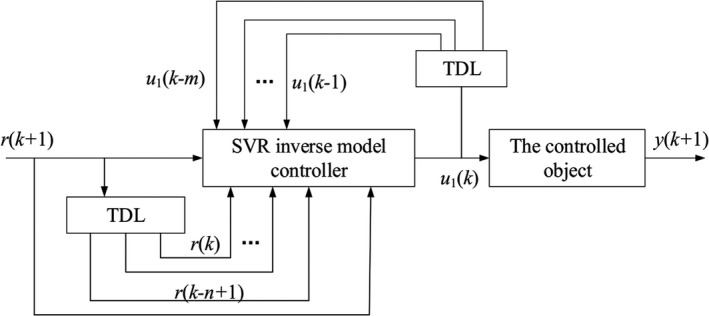
Structure of the inverse model controller based on support vector machines for regression

The inverse model controller can be realized by combining the trained SVR inverse model with the controlled object. The inverse model controller structure based on SVR is shown in Figure 7 (when *α* = 1), where *y* is the output of the controlled object, *u*_1_ is the predicted output of the SVR inverse model *g*_svr_, and *r* is the target signal.

The output expression of the SVR inverse model is shown in (7):

(7) $$u_{1}\left(k\right)=g_{\mathrm{svr}}\left[r\left(k+1\right),r\left(k\right),...r\left(k+1-n\right),u_{1}\left(k-1\right),...,u_{1}\left(k-m\right)\right].$$

The actual output of the controlled drying system is as follows:

(8) $$y\left(k+1\right)=f[y\left(k\right),...y\left(k+1-n\right),u_{1}\left(k\right),...,u_{1}\left(k-m\right)].$$

As seen from Figure 7, the SVR inverse model is directly connected with the controlled object, which is an open‐loop control. The open‐loop control system cannot handle the effect of the external disturbances; therefore, the dynamic performance and robustness of the direct inverse model controller need to be further improved. In actual control, the most effectively improvement method is to design a closed‐loop system by introducing the feedback control.

### 3.3 Design and optimization of the GO‐SVR‐IMCPID controller

**Figure 8:**
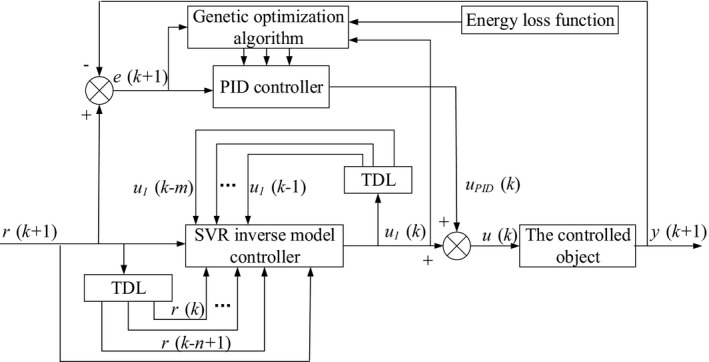
The structure of the GO‐SVR‐IMCPID

Aiming at the shortage of the direct inverse model controller, in this section, the PID control is added to the SVR inverse model controller, making the open‐loop control turn into a closed‐loop controller. In addition, the performance index function is designed by considering the equation of the energy loss and the dried grain quality. And to achieve the best controller effect, a genetic algorithm is used to optimize the controller parameters based on the performance index function. Thus, the controller performance will be improved by combining the inverse model control of SVR, the PID control, and the genetic optimization algorithm. The proposed controller is called for short GO‐SVR‐IMCPID in this paper, of which the structure is shown in Figure 8, where *u*_1_ is the output of the SVR inverse model *g*_svr_, *u*_PID_ is the output of the PID controller, *u* is the superposition of *u*_1_ and *u*_PID_, and *e* is the error between *y* and *r*.

When *k* ≥ *m* + 1, the output of the PID controller is shown in (9):

(9) $$u_{\text{PID}}\left(k\right)=u_{\text{PID}}\left(k-1\right)+k_{p}\Delta e\left(k\right)+k_{i}e\left(k\right)+k_{d}\left[\Delta e(k)-\Delta e(k-1)\right]$$

where

(10) e(k)=r(k)-y(k)

(11) Δe(k)=e(k)-e(k-1).

The control input of the controlled object is shown in (12):

(12) $$u\left(k\right)=u_{1}\left(k\right)+u_{\text{PID}}\left(k\right).$$

The output of the controlled object is shown in (13):

(13) y(k+1)=f[y(k),...,y(k+1-n),u(k),...u(k-m)].

### 3.4 Parameter optimization of the GO‐SVR‐IMCPID controller based on genetic algorithm

Genetic algorithm (GA) is an efficient stochastic search algorithm for solving optimization problems in artificial intelligence. It mimics the process of biological evolution by the processes of selection, crossover, and mutation, and is used to search the optimal solutions with the minimum fitness function value in the population of each iteration.

In this design, the PID parameters *k*p, *k*i, and *k*d are optimized to improve the control process by using the GA, of which the ranges are within [−10 10]. The performance objective function of grain drying is the minimum of the function *J*, which is established from the two aspects of decreasing the efficient energy consumption and improving the dried grain quality shown in (14):

(14) $$\text{Min}\left(J\right)=\left\{\begin{array}{cc} \sum_{k=1}^{N-1}\left[w_{1}\left|e(k)\right|+w_{2}u^{2}\left(k\right)\right]+w_{3}*t_{r}+w_{4}*E, & \text{if}\left|\text{erry}(k)\right|\leq \xi \\ \sum_{k=1}^{N-1}\left[w_{1}\left|e(k)\right|+w_{2}u^{2}\left(k\right)+w_{5}\left|\text{erry}(k)\right|\right]+w_{3}*t_{r}+w_{4}*E, & \text{if}\left|erry(k)\right|>\xi \end{array}\right.$$

where *e* means the input error; *N* is the total sampling event number; *t*_r_ means the rising time; *w*_1_, *w*_2_, *w*_3_, *w*_4_, and *w*_5_ are the weights; *u* means the output of the controller; *y*(*k*) is the output of the controlled system and the output error erry(*k*)= *y *(*k*) − *y*(*k *− 1); *E* is the total energy consumption of all sampling events shown in (15); the penalty function is used to avoid overshoot (*w*_5_ » *w*_1_); and ξ is the size of the overshoot to be controlled.

(15) $$E=\sum_{k=1}^{N}h_{g}*V_{g}*\left(\bar{M_{k}}\;-\bar{M_{i}}\right).$$

In (15), $\bar{M_{k}}\;$ is the average outlet grain MC of the *k*th sampling event of the drying section, decimal (wet basis, wb); $\bar{M_{i}}$ is the average inlet grain MC of the drying section, decimal (wb).

**Figure 9:**
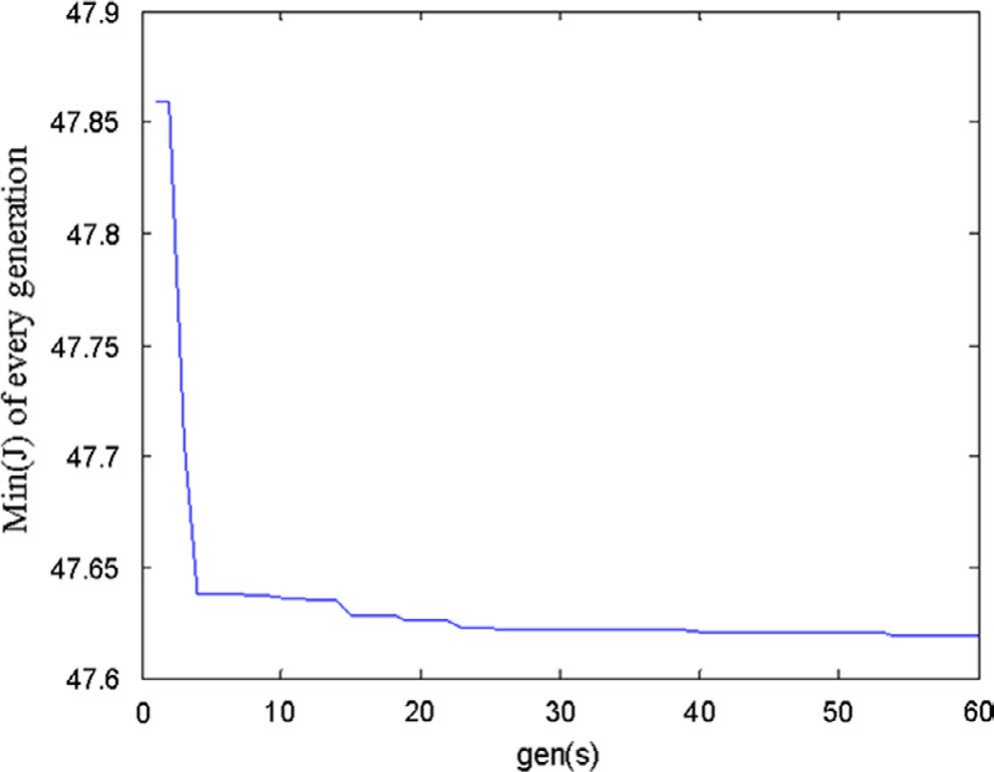
The fitness curve of simulation in every generation

In addition, the initial optimal parameter values by the GA are as follows: the generations of evolution: 60; the population size: 10; the individual number: 3; and the probability of selection, crossover, and mutation are respectively 0.9, 0.6, and 0.01. Figure 9 shows the fitness curve of simulation. After 60 generations of iteration, the optimal value of *J* is equal to 47.6, and the optimal parameters are equal to −5.0769, −9.9255, and −0.7240, respectively.

## 4 EXPERIMENTAL RESULTS AND ANALYSIS

### 4.1 Simulation experiment initialization

In simulation, assuming the model errors are zero, the continuous mixed‐flow drying model based on the Equation (1) is used to verify the effectiveness of the designed controller. The discharging grain flow rate of the dryer is taken as the control variable, and the outlet grain moisture of the drying section is taken as the controlled variable. In a certain time and drying condition, the hot air temperature, the airflow, and the initial grain temperature and moisture are not changed basically, so the variations of which can be regarded as the interferences in the control of grain dryer.

In this paper, we adopted the *ε*‐SVR type as the inverse model of which the parameter value*s δ a*nd *ε* are respectively 1,200 and 0.001, the width parameter *γ* of the adopted RBF function is equal to 2, and the simulation time is 200 sampling events. The *ε*‐SVR inverse model is trained with the training sample *D*_2_ which is obtained by the identification experiment. The constructed sampling data structure is as follows:

(16) $$\left\{\begin{array}{c} D_{2}=\left\{\left(X(k-2\right)\right.,Y\left(k-2\right)),k=3,...,199\}\\ X(k-2)=\{y(k+1),y(k),y(k-1),u(k-1),u(k-2)\}\\ Y(k-2)=u(k) \end{array}\right.$$

### 4.2 Modeling and generalization experiment of the inverse model based on SVR

**Figure 10:**
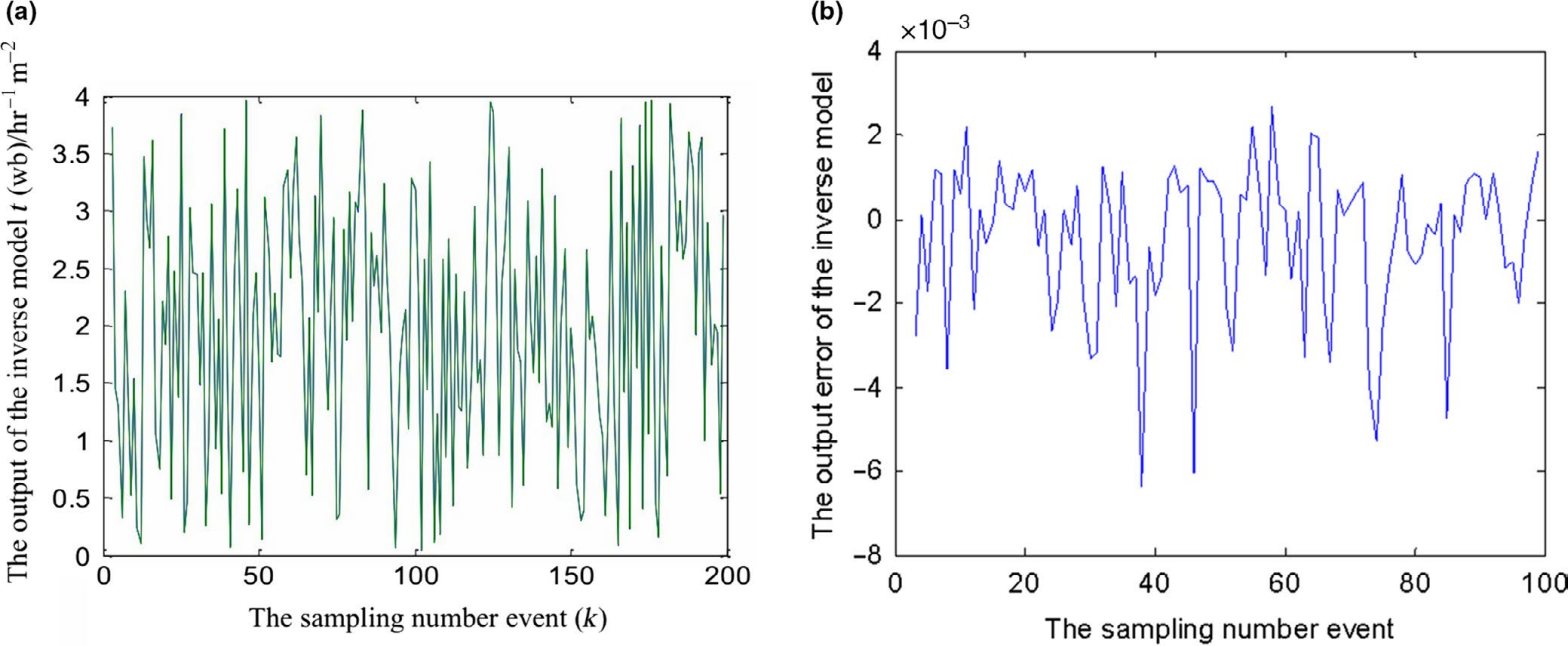
(a) The prediction simulation results of the identified support vector machines for regression (SVR) inverse model: the output. (b) The prediction simulation results of the identified SVR inverse model: the output relative error

In the identification experiment, the random noise input signal within a magnitude range of 0–4,000 kg hr^−1^ m^2^ is used to imitate different grain flow rates in the process of continuous drying. The system output is the final obtained outlet grain MC values of the drying section. The prediction simulation results of the identified SVR inverse model are shown in Figure 10, of which the output is the predicted grain flow rate in the requirement of different final outlet grain MC. It can be seen that the model errors are mostly within 10^–3^ order of magnitude, which shows that the SVR inverse model has a higher accuracy (*RMSE*: 0.038; *R*: 99.5%).

### 4.3 Control simulation experiment results

#### 4.3.1 The simulation results of the tracking control

**Figure 11:**
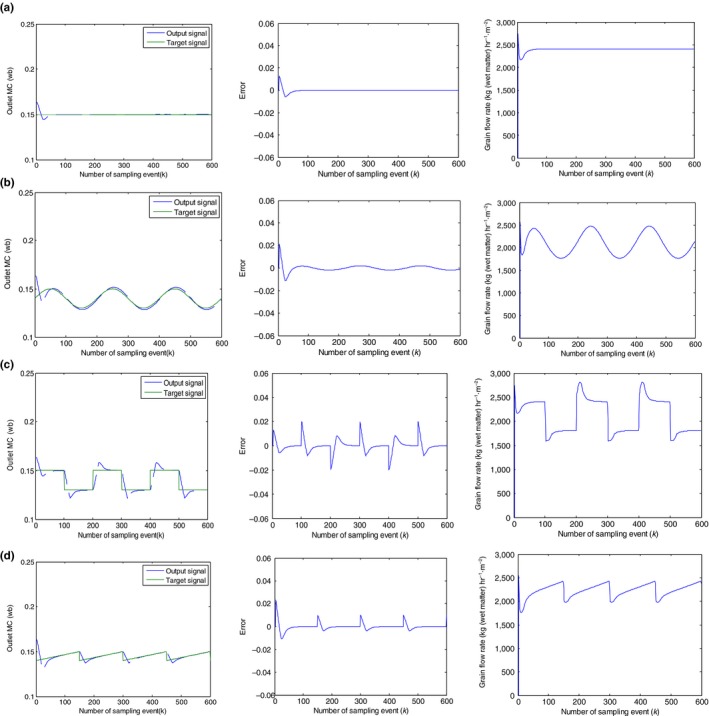
(a) The tracking control results of the control system: To the step signal. (b) The tracking control results of the control system: To the sinusoidal signal. (c) The tracking control results of the control system: To the square wave signal. (d) The tracking control results of the control system: To the sawtooth signal

In the tracking control simulation, the sample number is 600, the initial grain moisture is 23% (wb), the temperature of the hot air is equal to 100°C, and the tracked input signals are respectively the step signal with a magnitude of 15% imitating the final controlled outlet MC value, the sinusoidal signal, the sawtooth signal, and the square wave signal. In addition, the initial grain flow rate is set to 3,000 kg hr^−1^ m^2^, and the grain flow rate is controlled by the proposed controller at the sampling event which is equal to the dwell time in a bed element. The controlled object is the outlet grain MC of the drying section. According to the drying model simulation experiment based on the Equation (1), the drying section is supposed to be equal to 20 bed elements. By connecting the established inverse model with the original controlled system in series and combining with the genetically optimized PID output feedback controller, the simulations of tracking control in the various input signal have been tested shown in Figure 11.

As seen from Figure 11a, in the tracking control to the step signal, the GO‐SVR‐IMCPID can rapidly and steadily adjust the output to the target value of 15% by controlling the grain flow rate at every sampling event, of which the overshoot is smaller, the output errors are mostly controlled within the range of 0.001 in about 50 sampling intervals, and the final steady‐state errors are controlled within 10^–13^ order of magnitude. Furthermore, it can be seen from Figure 11b–d, under the other three tracking signals, the proposed controller also has excellent tracking performances, of which the output can be rapidly adjusted to the target value with a good precision of control. Hence, the tracking control performances under four typical target signals show that the proposed GO‐SVR‐IMCPID has a good dynamic performance with smaller overshoot, shorter rising and adjusting time, and high accuracy. The tracking control ability under four typical signals demonstrates the efficiency of the proposed controller for the nonlinear grain drying process.

#### 4.3.2 The anti‐interference test results

To access the anti‐interference control performance of the proposed controller, we adopted two‐step disturbance signals imitating sudden changes in the outlet grain MC caused by the effect of the interference signals, one of which is with a magnitude of 0.04 at the sample event of 100, and the other of which is with a magnitude of −0.04 held for 30 sampling events at the sample event of 150.

**Figure 12:**
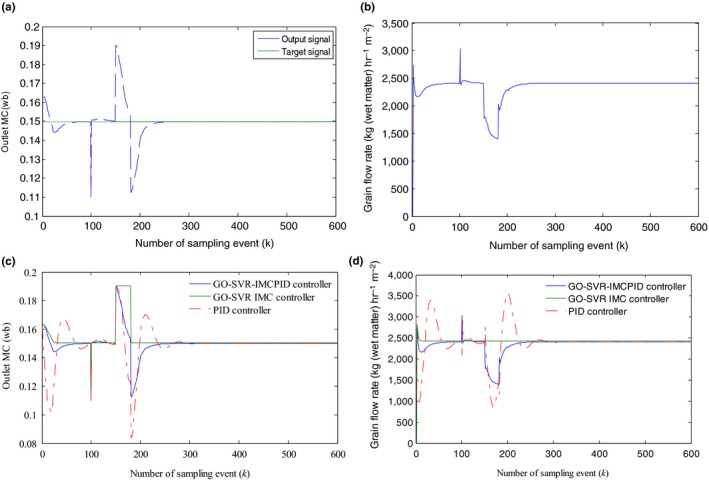
(a) The anti‐interference simulation results of the GO‐SVR‐IMCPID: the system response for the two disturbances at the sampling event *k* = 100 and *k* = 150. (b) The anti‐interference simulation results of the GO‐SVR‐IMCPID: control signal‐the grain flow rate. (c) The anti‐interference simulation results of the GO‐SVR‐IMCPID: the anti‐inference simulation comparison results with the other signals. (d) The anti‐interference simulation results of the GO‐SVR‐IMCPID: control signal comparison of different controllers

The anti‐interference simulation results are shown in Figure 12a,b, and the simulation comparison results with the PID controller and the SVR‐IMC controller are shown in Figure 12c,d.

It can be seen from Figure 12a that the output of the GO‐SVR‐IMCPID control system can be adjusted to the target value rapidly and steadily when the disturbances exist, and the fluctuations caused by the disturbances can be rejected rapidly, and the steady errors are smaller, showing an excellent anti‐interference ability. Figure 12b shows the GO‐SVR‐IMCPID controller can response to the first and the second output disturbance rapidly by decreasing and increasing the grain flow rate to a suitable value, respectively. Furthermore, as seen from Figure 12c, it can also be seen that the GO‐SVR‐IMCPID controller is better to the other two compared controllers according to the control performance comparison when an interference exists during the drying process; For the same test, the GO‐SVR‐IMC controller has no ability to restrain the effect of the output disturbances because of the open‐loop control structure, although it can achieve an accurate tracking control in the absence of interference. For the PID controller, it can suppress the effect of the disturbances, but the fluctuations caused by the disturbances are larger than that of the GO‐SVR‐IMCPID controller. Figure 12d shows the control signal (the grain flow rate) output comparison of different controllers, from which it can be seen that the GO‐SVR‐IMCPID controller performs well, while there is no change in the grain flow rate of the GO‐SVR‐IMC when the inference comes, and the fluctuations of the control signal of the PID controller is larger than the GO‐SVR‐IMCPID. The simulation results show that the GO‐SVR‐IMCPID has improved the control performance of the GO‐SVR‐IMC controller and the PID controller, and it performs best under uncertainty output disturbances circumstances.

#### 4.3.3 The robustness test results

**Figure 13:**
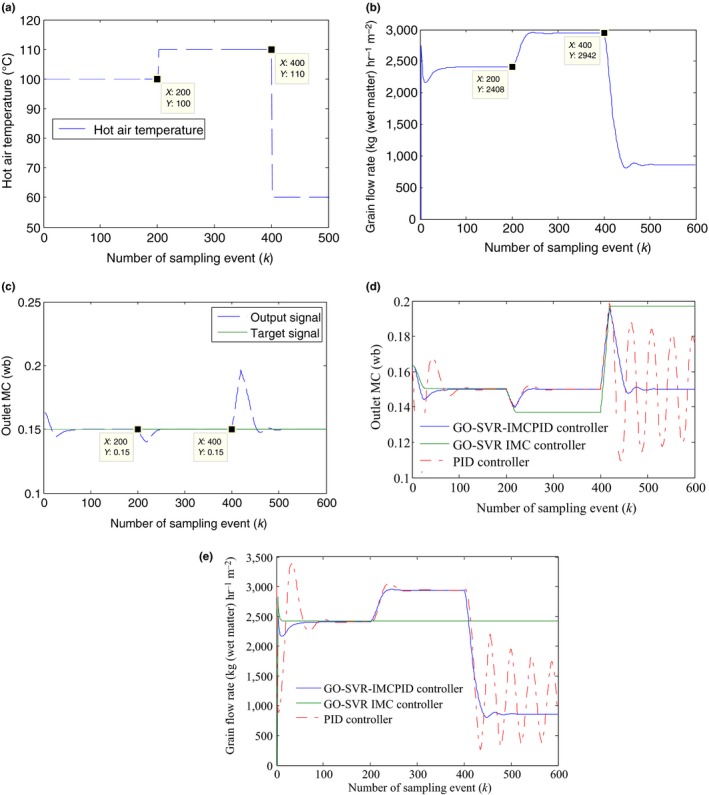
(a) The first robustness test results to the change of the hot air: the hot air temperature change. (b) The first robustness test results to the change of the hot air: the outlet grain moisture content of the drying section. (c) The first robustness test results to the change of the hot air: the change of control signal—the grain flow rate. (d) The first robustness test results to the change of the hot air: the robust test comparison of different controllers. (e) The first robustness test results to the change of the hot air: the control signal comparison of different controllers

To evaluate the robustness of the proposed controller when the system parameters were changed, two robustness tests were carried out, one of which was to study the response ability of the system to the variation of the drying hot air, the other of which was to investigate the response ability of the system to the change of the initial inlet grain MC. Suppose that the temperature of hot air at 200 sampling points suddenly rises to 110°C and then dropped to 60°C after the 400th sampling points shown in Figure 13a, the first robustness simulation results to the change of the hot air are shown in Figure 13b,c. Figure 13d,e shows the simulation result comparison of the first robustness test with the other two controllers.

**Figure 14:**
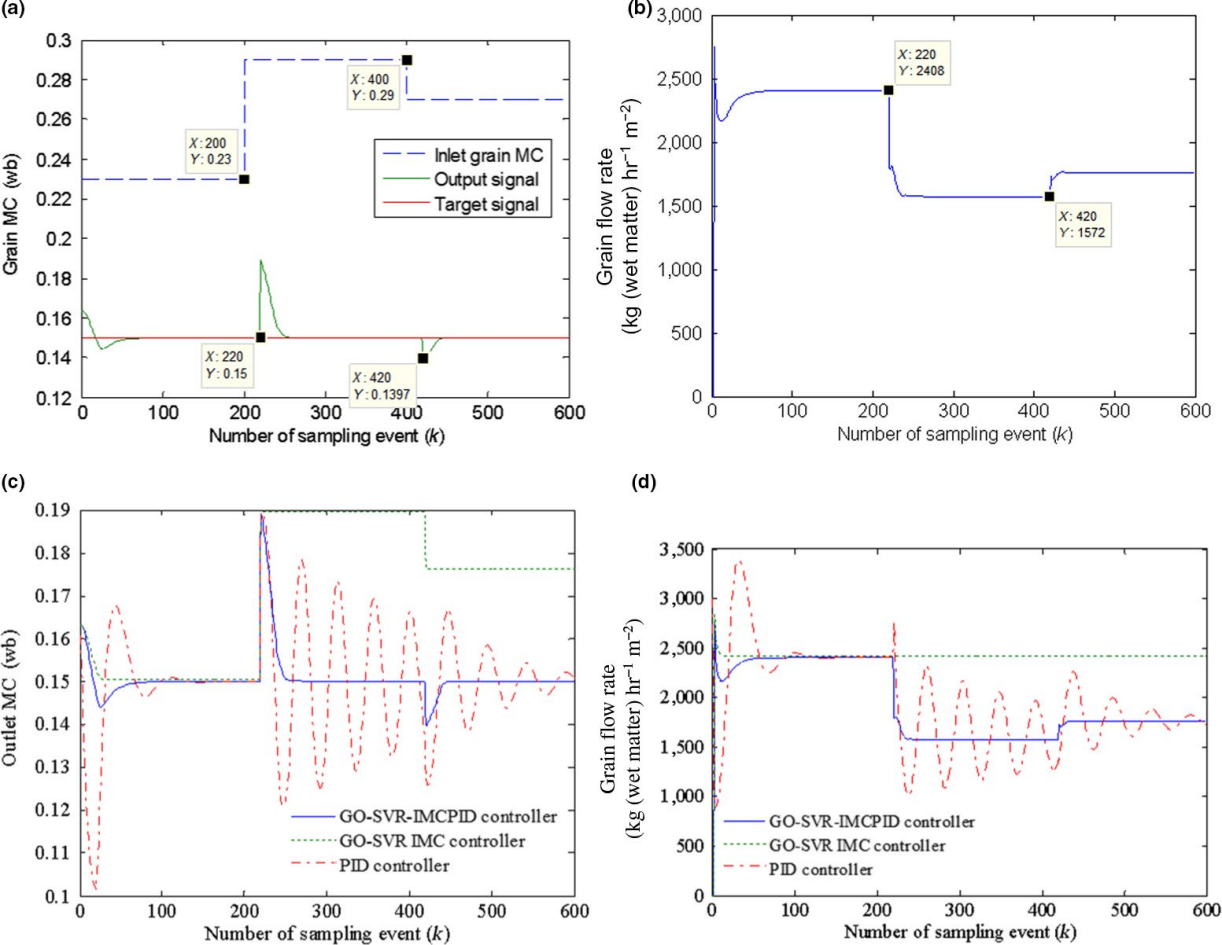
(a) The second robustness test results to the change of the initial inlet grain moisture content (MC): the inlet grain MC change and the system response result. (b) The second robustness test results to the change of the initial inlet grain MC: the change of control signal—the grain flow rate. (c) The second robustness test results to the change of the initial inlet grain MC: the robust test comparison of different controllers. (d) The second robustness test results to the change of the initial inlet grain MC: the control signal comparison of different controllers

Figure 14a,b shows the second robustness simulation results when the initial inlet moisture suddenly rose to 28% (wet base) at the 200th sampling points and then dropped to 27% (wb) after the 400th sampling points, and Figure 14c,d shows the simulation result comparison of the second robustness test with the other two controllers.

The first robustness test analysis is as follows: It can be seen from Figure 13b that the GO‐SVR‐IMCPID controller can make an immediate response to the change of the hot air temperature to counteract the disturbance effect by increasing the grain flow rate after the 200th sampling event and by decreasing the grain flow rate after the 400th sampling event. As shown in Figure 13c, the outlet grain MC can be rapidly adjusted to the steady target value again when the hot air temperature changes. As seen from Figure 13d, the GO‐SVR‐IMCPID controller performs best with a rapid response speed, smaller overshoot, and good robustness performance among the compared three controllers; however, for the same robustness test, the GO‐SVR‐IMC controller cannot respond to adjust the system output to the target value when the hot air temperature is changed; although the PID controller manages to handle the first variation of the hot drying air after the 200th sampling event, it cannot resist the effect of the second change of hot drying air, showing an undesirable oscillatory behavior after the 400th sampling event. Figure 13e compares the output of the grain flow rate generated by the three controllers, which shows that the control signal of the GO‐SVR‐IMCPID can be adjusted to realize the tracking control of the target value with the change of the hot air temperature; however, the control signal of the GO‐SVR‐IMC controller has no response to the variation of the hot air temperature, and the control signal of the PID controller shows an oscillatory behavior in the second variation of the hot air temperature.

The second robustness test is as follows: As seen in Figure 14a, to avoid overdrying or insufficient drying, the control signal does not change immediately when the initial inlet grain MC changes, but begins to change when the unaffected grains in the drying section have been evacuated which is equal to 20 thin drying beds according to the model simulation verification. It shows that the GO‐SVR‐IMCPID controller can rapidly adjust the output back to the desired outlet MC after 20 sampling events when the initial grain MC changes and the output errors affected by the disturbance are smaller. It can be seen from Figure 14b that the control signal of the GO‐SVR‐IMCPID controller firstly goes down to resist the effect caused by the initial grain MC increase at the 220th sampling time and then rises up to resist the effect caused by the initial grain MC decrease after the 420th sampling time. From the comparison of the three controllers shown in Figure 14c,d, it can be seen that the GO‐SVR‐IMC controller and the PID controller show an unsatisfactory control performance in handling the disturbance, of which the system output cannot be adjusted to the target value steadily when the initial grain MC changes, and the grain flow rate of the GO‐SVR‐IMC controller has no response to the initial grain MC changes. And the grain flow rate of the PID controller shows a bigger oscillatory behavior in handling the disturbance.

In addition, from the simulation results of Figures 12, 13, 14, it can also be seen that the PID controller has a larger overshoot in tracking the target value than the GO‐SVR‐IMCPID controller at the beginning of the target signal.

#### 4.3.4 Comparison with the previously designed GO‐SVR‐IMPC controller

**Figure 15:**
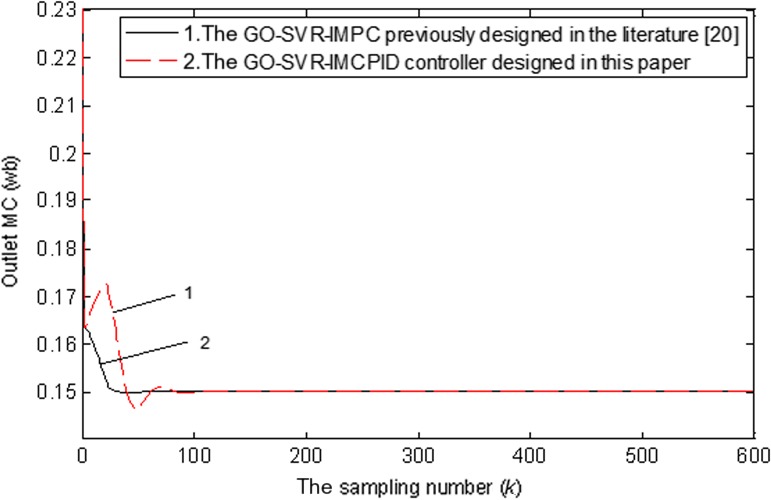
The tracking control performance comparison of two kinds of support vector machines for regression controller

**Figure 16:**
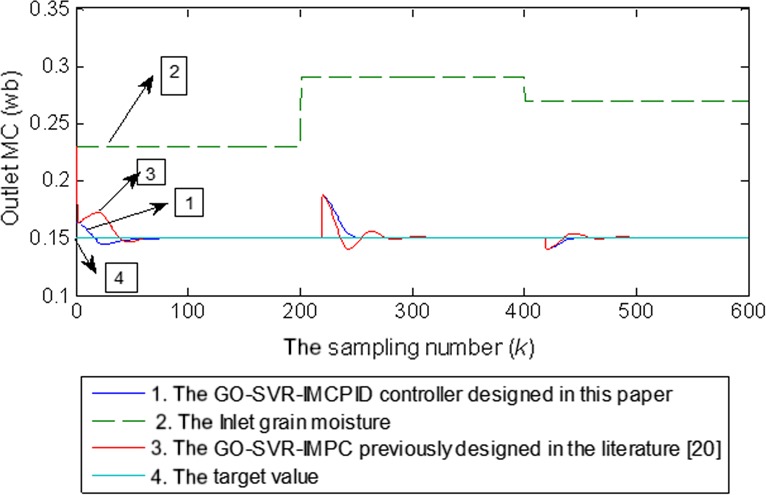
The robustness control performance comparison of two kinds of support vector machines for regression controller

The tracking control and the robustness test performances with GO‐SVR‐IMPC previously designed in another paper (Dai et al., 2018 are compared when the inlet grain MCs suddenly change. The comparison results are shown in Figures 15 and 16, respectively. From the comparison results, it can be seen that the control effects of the two controllers are both excellent. Among them, the GO‐SVR‐IMCPID controller designed in this paper has better control performance than the GO‐SVR‐IMPC. It has shorter time to reach the target value and smaller overshoot in tracking control of the target value. For the GO‐SVR‐IMCPID controller, when the inlet MCs of grain in the machine suddenly change, the time to adjust the output to the target value is shorter and the fluctuation is smaller. Based on the same performance objective function, the performance index *J* value of GO‐SVR‐IIMCPID controller is equal to 46.7, which is 18.8% smaller than that of GO‐SVR‐IMPC controller, which is equal to 57.5.

In all, in this study, the effectiveness of the GO‐SVR‐IMC controller in controlling the complex grain drying process has been demonstrated by the tracking control, the antidisturbance test, two robustness tests, and the control performance comparison with the other compared controllers.

## 5 CONCLUSION

In this paper, an intelligent controller (GO‐SVR‐IMCPID) based on SVM modeling method for a grain dryer is proposed. It combines inverse model control algorithm, traditional PID algorithm, and genetic optimization algorithm. The inverse model control is an open‐loop control method, which is simple and feasible in the situation of no disturbances, and it forms a pseudolinear system by connecting the inverse system to the original system. However, there are two drawbacks, one of which is that the controller has no ability to handle the external disturbance, and the other of which is that the accuracy of the controller depends on the modeling precision of the inverse model. In this paper, a closed‐loop IMC optimization controller based on SVR is designed by introducing PID feedback controller, SVR modeling method, and GO algorithm from the perspective of energy consumption and dried grain quality. To verify the feasibility of the GO‐SVR‐IMCPID in controlling the grain dryer, a nonlinear math model of continuous mixed‐flow drying process has been adopted to verify the control ability of the controller. The simulations of tracking control, anti‐interference test, and robustness test have been made by programming in MATLAB. The simulation results show that the GO‐SVR‐IMCPID has good tracking control performance, strong ability of anti‐interference, and good robustness performance. As seen from the simulation results, although the SVR inverse model controller (GO‐SVR‐IMC) has good tracking control performance under the condition of no interference, it has no ability to cope with the effect of external disturbances and parameter changes, in addition, although the PID controller can deal with some interferences, the fluctuations caused by the disturbances are bigger; furthermore, it cannot handle the effect of such parameter changes as the hot air or the inlet moisture MC, showing some unsatisfactory oscillatory behavior. By comparing with different controllers including the previously designed GO‐SVR‐IMPC controller, the experimental results have demonstrated that the GO‐SVR‐IMCPID has excellent control performances in controlling the complex grain drying process. It provides a good reference for the field control of grain dryer.

## CONFLICT OF INTEREST

The authors declare that there is no conflict of interest in this paper.

## ETHICAL APPROVAL

All authors have made equally substantial contributions to the writing of the paper and will be jointly and severally liable for the contents of the manuscript. The authors declare that there is no conflict of interest in this paper. Human and animal study is unnecessary to our study; thus, no ethical approval and patient consent are required.

## Data Availability

The mathematical model identification data are collected from the practical drying experiment, and the control experimental result dataset was analyzed in the study based on the drying model (1) in MATLAB, which are available from the first and corresponding author on reasonable request.
